# *Shewanella* spp. Genomic Evolution for a Cold Marine Lifestyle and *In-Situ* Explosive Biodegradation

**DOI:** 10.1371/journal.pone.0009109

**Published:** 2010-02-08

**Authors:** Jian-Shen Zhao, Yinghai Deng, Dominic Manno, Jalal Hawari

**Affiliations:** Biotechnology Research Institute, National Research Council Canada, Montreal, Quebec, Canada; Texas A&M University, United States of America

## Abstract

*Shewanella halifaxensis* and *Shewanella sediminis* were among a few aquatic γ-proteobacteria that were psychrophiles and the first anaerobic bacteria that degraded hexahydro-1,3,5-trinitro-1,3,5-triazine (RDX). Although many mesophilic or psychrophilic strains of *Shewanella* and γ-proteobacteria were sequenced for their genomes, the genomic evolution pathways for temperature adaptation were poorly understood. On the other hand, the genes responsible for anaerobic RDX mineralization pathways remain unknown. To determine the unique genomic properties of bacteria responsible for both cold-adaptation and RDX degradation, the genomes of *S. halifaxensis* and *S. sediminis* were sequenced and compared with 108 other γ-proteobacteria including *Shewanella* that differ in temperature and Na^+^ requirements, as well as RDX degradation capability. Results showed that for coping with marine environments their genomes had extensively exchanged with deep sea bacterial genomes. Many genes for Na^+^-dependent nutrient transporters were recruited to use the high Na^+^ content as an energy source. For coping with low temperatures, these two strains as well as other psychrophilic strains of *Shewanella* and γ-proteobacteria were found to decrease their genome G+C content and proteome alanine, proline and arginine content (p-value <0.01) to increase protein structural flexibility. Compared to poorer RDX-degrading strains, *S. halifaxensis* and *S. sediminis* have more number of genes for cytochromes and other enzymes related to RDX metabolic pathways. Experimentally, one cytochrome was found induced in *S. halifaxensis* by RDX when the chemical was the sole terminal electron acceptor. The isolated protein degraded RDX by mono-denitration and was identified as a multiheme 52 kDa cytochrome using a proteomic approach. The present analyses provided the first insight into divergent genomic evolution of bacterial strains for adaptation to the specific cold marine conditions and to the degradation of the pollutant RDX. The present study also provided the first evidence for the involvement of a specific *c*-type cytochrome in anaerobic RDX metabolism.

## Introduction

The oceans and their sediments have long been a sink for wastes from numerous human activities near shore and on the open ocean. Undersea unexploded ordnances (UXO) [1]–[3] are a source of hexahydro-1,3,5-trinitro-1,3,5-triazine(RDX), 2,4,6-trinitrotoluene (TNT), and dinitrotoluene (DNT) which are toxic to humans and other organisms[4]–[6]. *Shewanella* are ubiquitous in surface, coastal, and deep sea water as well as in sediments such as the highly polluted Baltic Sea and the coastal area of North Atlantic (Table 1). Some strains were also found in lake and groundwater environments (Table 1). *Shewanella* can grow anaerobically using nitrate, manganese dioxide (MnO_2_), trimethylamine *N-*oxide (TMAO), and/or dimethyl sulfoxide (DMSO) commonly found in marine sediment environments, as terminal electron acceptors [7]–[9]. *Shewanella* are well known for their ability to oxidize organic matter and reduce chlorinated pollutants [10], as well as metal ions including Fe (III) and uranium (VI) [7], [8], [11]. Recently, strains of *Shewanella* were found to be dominant (8.8% of total cultured bacteria) in a historical UXO-dumping site, Emerald Basin [12], [13], a 250 m deep depression on the continental shelf, and 60 nautical miles south of Halifax, Nova Scotia. Two representative strains were shown to be capable of degrading RDX [12], TNT, DNT, perchlorate (Jian-Shen Zhao et al, unpublished results), and nitrate commonly present in UXO. Subsequent characterizations of these strains revealed that they represent two new species and consequently were designated *Shewanella sediminis* HAW-EB3 [14] and *Shewanella halifaxensis* HAW-EB4 [15]. New evidence also showed that incubation of sediment with nitrated compounds such as 2,4-DNT led to enrichment of *Shewanella* [16]. Although several aerobic RDX-degrading were isolated [17], none of the strains were sequenced for their genomes. *S. halifaxensis* and *S. sediminis* were the first anaerobic RDX-mineralizing bacteria known to be dominant in a contaminated UXO site. In the present study, the genomes of the two strains of *Shewanella* along with two most closely related reference strains not from this contaminated site, *Shewanella woodyi* [18] and *Shewanella pealeana* [19], sequenced by the Joint Genomic Institute (JGI) of United States, were compared to determine their novel genomic properties.

**Table 1:** Phenotypic properties of *Shewanella* used for comparative genomic analyses.

Strains	symbol	16S rDNA/ genome accession	Cluster	Biodegradation^§^		Requirement			Site of isolation
				RDX rate	NA	NaCl	OT	G30	
*S. halifaxensis* HAW-EB4 [15]	ha	AY_579751 / CP_000931	I	8.1	+	+	10	–	Deep cold sediment, North Atlantic
*S. sediminis* HAW-EB3 [14]	se	AY_579750 / CP_000821	I	13	+	+	10	–	Deep cold sediment, North Atlantic
*S. pealeana* ATCC 700345 [19]	pe	AF_011335 / CP_000851	I	3.8	+	+	25	–	Squid nidamental gland, North Atlantic
*S. woodyi* ATCC 51908 [18]	wo	AF_003459 / CP_000961	I	2.5	+	+	25	–	Deep Alborane sea, Mediteranean
*S. loihica* PV-4 ATCC BAA1088 [27]	lo	AF_387348 / CP_000606	I	ND	ND	+	18	+(42)	seawater near active sea vent, Hawaii , Pacific
*S. frigidimarina* NCIMB 400 [25]	fri	Y_13699 / CP_000447	II	ND	ND	–	20–22	–	near Aberdeen, UK, North sea of Atlantic
*S. denitrificans* DSM15013 (OS217) [70]	de	AJ_311964 / CP_000302	II	ND	ND	–	20–25	–	120–130 m sea water, Baltica
*S. baltica* ATCC BAA-1091 (OS155) & plasmids[71]	ba5	CP_000563, CP_000567	II	ND	ND	–	ND	+	sea water, Baltic
*S. baltica* OS185 & plasmid [71]	ba8	CP_000753, CP_000755	II	ND	ND	–	ND	+	sea water, Baltic
*S. baltica* OS195[71]	ba9	AJ_000216 / CP_000891, CP_000894	II	ND	ND	–	ND	+	sea water, Baltic
*S. amazonensis* ATCC 700329 (SB2B )[26]	am	AF_005248 / CP_000507	II	ND	ND	–	37	+(45)	Low salinity marine mud, Amazon delta, Atlantic
*S. oneidensis* MR-4^¶^	on4	AF_005252 / CP_000446	II	ND	ND	–	ND	+	Shalow water, Black Sea
*S. oneidensis* MR-7^¶^	on7	AF_005253 / CP_000444, CP_000445	II	ND	ND	–	ND	+	Shalow water, Black Sea
*S. putrefaciens* W3-18-1^¶^	pw3	AF_387350 / CP_000503	II	ND	ND	–	ND	+	sediment, 997 m, Pacific
*S. oneidensis* MR-1 (ATCC 700550)[9]	on1	AF_005251 / AE_014299, AE_014300	II	+	+	–	30	+(40)	Freshwater lake, Oneida Lake
*S. putreficiens* CN-32 ^¶^	pcn	CP_000681	II	ND	ND	–	ND	+	subsurface ground water
*Shewanella* sp. ANA-3^¶^	an	AF_136392 / CP_000469, CP_000470	II	ND	ND	–	ND	+	Brackish estuary, woods hole, North Atlantic

Note: Data were from the references given in the strain column, present study (§) or from Drs Jim Fredrickson and Margaret Romine (¶). RDX rates, nM h^−1^(*S. hanedai* ATCC 33224, 1.5); NA, dinitrotoluenes and trinitrotoluene; OT, optimal temperature (°C); G30, growth at 30°C [In brackets are the maximal growth temperature]; ND, no data; +, positive; –, negative.

**Figure 1:**
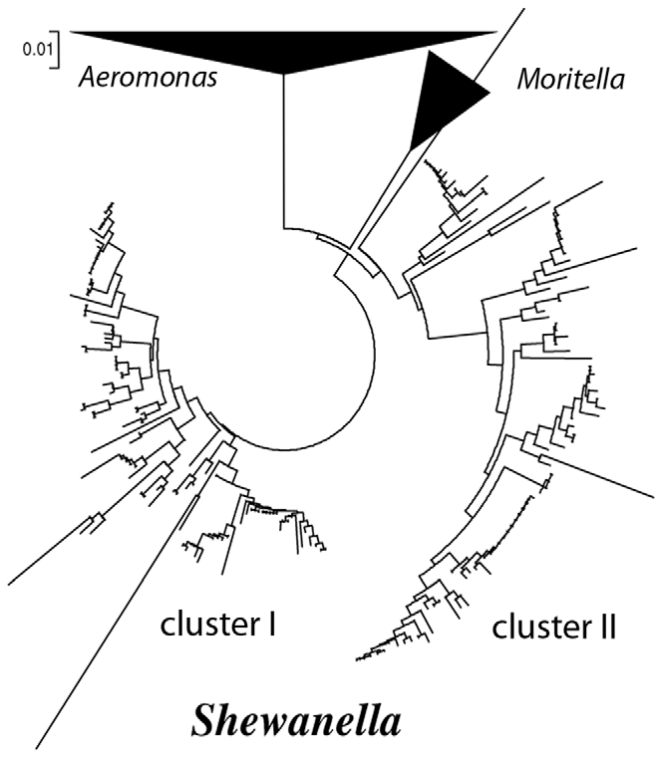
Phylogenetic analysis of partial 16S rDNA sequences of all cultivated and uncultivated *Shewanella*. The sequences of *Shewanalla*, *Aeromonas* and *Moritella* were all from GenBank (362 taxa in total). The phylogenetic tree was generated based on pair-wise nucleotide distance of Kimura two-parameter using the neighbour-joining method (pair-wise deletion, 3000 bootstrap value) included in the MEGA3 software package [68].

**Figure 2:**
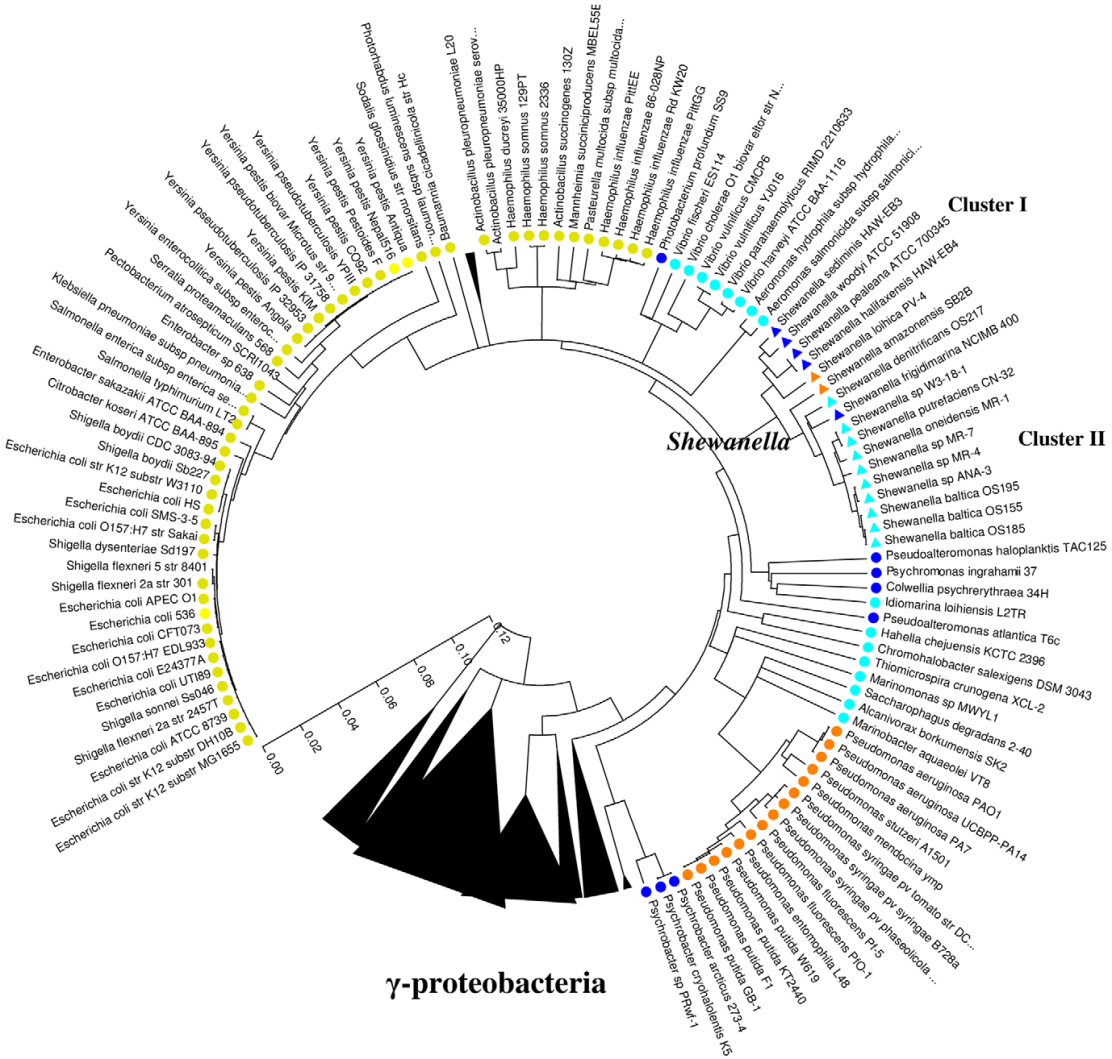
Phylogenetic analysis of the complete 16S rDNA sequences of γ-proteobacteria. Only the 110 strains compared for their genomes were listed. Yellow (coliforms) and orange (soil *Pseudomonas*) colors indicate bacteria living in warmer environments. Blue colors indicate γ-proteobacteria living in colder marine/aquatic environments. Two 42°C -tolerant *Shewanella* are indicated by orange triangles. The dark blue colors indicate bacteria adapted to deep cold sea and polar environment. The phylogenetic tree was generated based on pair-wise nucleotide distance of Kimura two-parameter using the neighbour-joining method (complete deletion, 3000 bootstrap value) included in the MEGA3 software package [68].

All species of *Shewanella*, isolates or environmental clones, fell into two major clusters based on their 16S rDNA sequences (Fig. 1) as well as phenotypic properties of isolates [14]. *Shewanella* retrieved from the deep sea, where temperatures are low (4–10°C) and the salt concentrations are high (4%), were included in cluster I. The other *Shewanella* from environments including shallow coastal area, ocean surface, freshwater lakes and subsurface groundwater were included in cluster II. The water temperature and/or salinity in the above environments varied depending on season, climate zone and depth (Table 1), but usually were higher in temperatures and/or lower in salinity as compared to the deep sea. *S. halifaxensis*, *S. sediminis, S. pealeana* and *S. woodyi* were members of cluster I *Shewanella* adapted to the colder and deeper part of marine eco-system; they required Na^+^ and preferred low temperatures for growth and thus considered as cold-adapted obligate marine *Shewanella* [14], [15]. Other 13 strains of *Shewanella* listed in Table 1, mostly distributed in cluster II and not found at the UXO-contaminated Halifax site, had no growth requirement for Na^+^ and low temperature. Most *Shewanella* in cluster II were considered as non-obligate marine and were available for genomic comparison at the beginning of this study (Table 1). Comparing genomes of obligate marine *Shewanella* in cluster I with non-obligate marine *Shewanella* in cluster II (as listed in Table 1) would provide general insight into bacterial evolution for cold and marine adaptation. The overall goals of the present study were to compare genomes of *S. halifaxensis* and *S. sediminis* with reference strains (listed in Fig. 2) in order to understand their genomic evolution pathways for adaptation to a UXO-contaminated cold marine sediment site as well as for in-situ degradation of explosive RDX.

## Results and Discussion

### *S. halifaxensis* and *S. sediminis* Genomic Evolution

**Figure 3:**
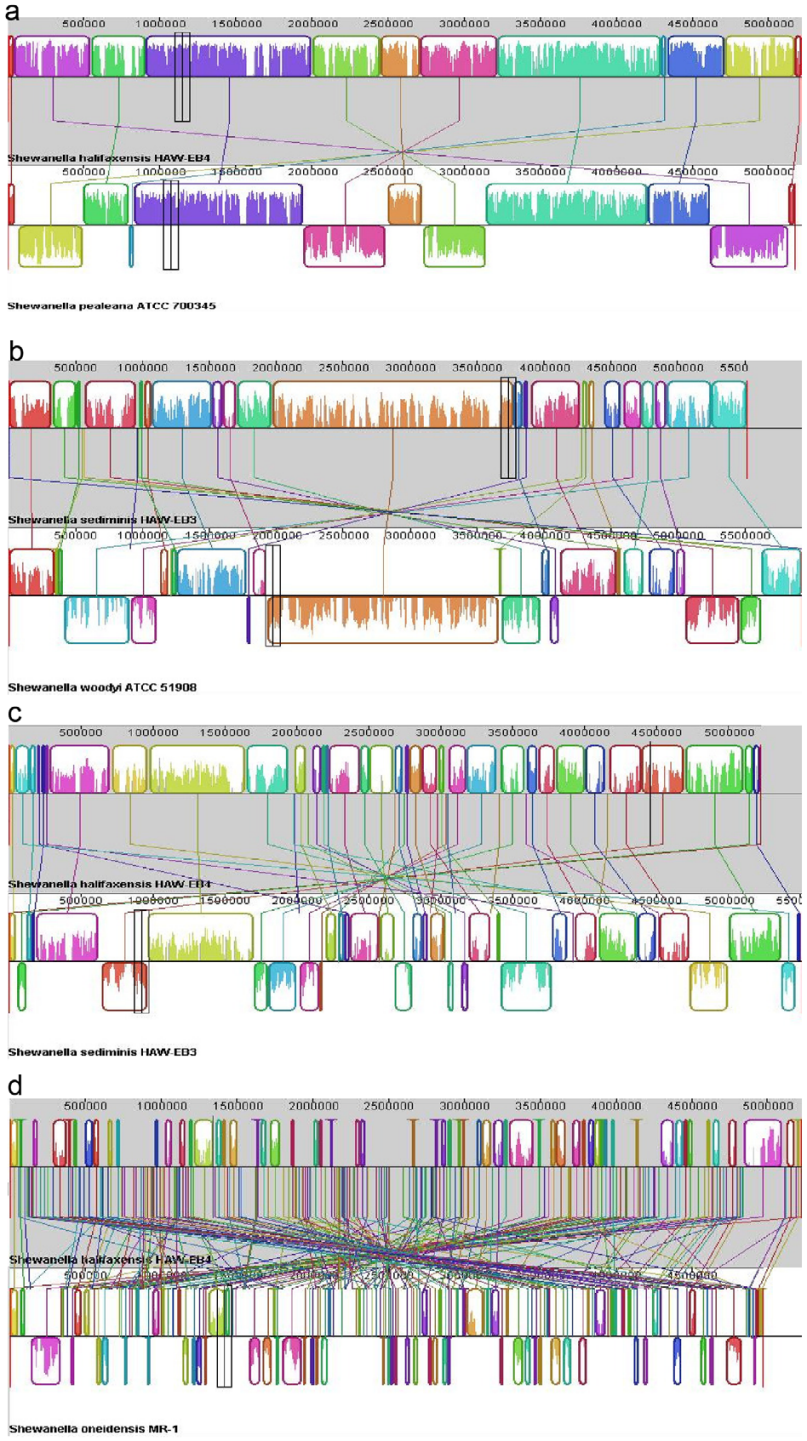
Whole-genome alignment of *S.halifaxensis* and *S. sediminis* with related *Shewanella.* Progressive-Mauve software 2.2.1 [63] was used to prepare the following alignment (minimal LCB weight, total number of LCBs). a), *S. halifaxensis/S. pealeana* (35774 base, 12); b) S. *sediminis/S. woodyi* (4207 base, 28); c) *S. halifaxensis/S. sediminis* (2693 base, 43); d) *S. halifaxensis/S. oneidensis* (118 base, 211). The same colored blocks indicate the segments, or Locally Collinear Blocks (LCBs) conserved among the two bacteria compared (linked by same line). Inverted LCBs are linked by lines crossing the mid point of chromosomes and appear on the opposite strand of the genome compared.

As shown in Figure 2, a comparison of the complete sequences of 16S rDNA genes of *S. halifaxensis* and *S. sediminis* with 15 other sequenced *Shewanella* revealed that the two marine bacteria were most closely related to Na^+^-requiring marine strains *S. woodyi* and *S. pealeana*. Pair-wise whole-genome alignment among the 17 sequenced *Shewanella* (Table 1) also demonstrated that the complete sequences of *S. halifaxensis* and *S. sediminis* were mostly conserved in *S. pealeana* (Fig 3a) and *S. woodyi* (Fig 3b). As shown in Figure 3a, *S. halifaxensis* had 10 very large genomic segments or Locally Collinear Blocks (LCB) ranging from 0.26 to 1.09 Mb conserved in *S. pealeana*. Gaps between LCBs were small and only five LCBS were inverted in *S. pealeana*. *S. halifaxensis* whole genome was also aligned very well with *S. sediminis* (Fig 3c) and *S. woodyi* (not shown). However the LCBs of the two pairs were shorter and more inversions occurred (*S. sediminis*, 30 LCBs, 0.06–0.67 Mb, 12 inversions; *S. woodyi*, 38 LCBs, 0.05–0.38 Mb, and 14 inversions) as compared to the alignment with *S. pealeana*. This demonstrates that *S. halifaxensis* genome is best conserved in *S. pealeana* than in *S. sediminis* and *S. woodyi*. In the case of *S. sediminis*, its whole genome was best aligned with *S. woodyi* with 9 large conserved LCBs (0.18–1.8 Mb) and 6 inversions (Fig 3b). In comparison, *S. sediminis* genome had relatively small LCBs conserved in genomes of *S. halifaxensis* and *S. pealeana* with more inversions (*S. halifaxensis*, 30 LCBs, 0.06 to 0.67 Mb, 15 inversions, Fig 3c; *S. pealeana*, 30 LCBs, 0.04–0.7Mb, 17 inversions). In contrast, genome of freshwater strain *Shewanella oneidensis* aligned very poorly with marine strain *S. halifaxensis* or *S. sediminis* with very small LCBs, large unaligned gaps between LCBs and many inversions (*S. halifaxensis*, 0.05 to 0.24 Mb, 43 inversions, Fig 3d; *S. sediminis*, 0.03 to 0.13Mb, 33 inversions). Poor whole-genome alignment was also observed between the two RDX-degrading marine *Shewanella* and other non-obligate marine *Shewanella* in cluster II (data not shown). This clearly demonstrates that the genomes of marine strains of *Shewanella* are significantly different from the genomes of freshwater and non-obligate marine strains of *Shewanella*.

To further determine the genomic similarity between the two RDX-degrading obligate marine *Shewanella* (*S. halifaxensis* and *S. sediminis*) and other reference bacteria living in similar or contrasting environments, the sequences of all their deduced proteins were compared using BLAST (cut-off E-value of e-20), to all 623 bacteria that were available for genomic comparison. The total deduced proteins of *S. halifaxensis* were found best conserved in *S. pealeana* (83.9%), whereas those of *S. sediminis* were best conserved in *S. woodyi* (69.6%) (Table 2). The total deduced proteins of both strains were much less conserved in non-obligate marine *Shewanella* (58–61%) such as *S. oneidensis*. This further demonstrates that the genomes of *S. halifaxensis* and *S. sediminis* have evolved along with *S. pealeana* and *S. woodyi* as an obligate marine lineage of *Shewanella*, distinct from those in cluster II.

**Table 2:** Genomic properties of four obligate marine species of *Shewanella* sequenced in the present study.

Genomic properties		*S. halifaxensis* HAW-EB4	*S. pealeana* ATCC 700345	*S. sediminis* HAW-EB3	*S. woodyi* ATCC 51908
General properties	Genome size (base pair)	5,226,917	5,174,581	5,517,674	5,935,403
	Gene count	4464	4438	4666	5096
	G + C content (%)	44.6	44.7	46.1	43.7
	Total CDS predicted	4278	4241	4512	4880
Number of CDS (% of total CDS) conserved in	*S. halifaxensis* (ha)	4278 (100)	3589	3088 (68.4)	2921
	*S. pealeana* (pe)	3589 (83.9)	4241 (100)	3061 (67.8)	2967
	*S. sediminis* (se)	3088 (72.2)	3061	4512 (100)	3133
	*S woodyi* (wo)	2921 (68.2)	2967	3133 (69.4)	4880 (100)
	*S. oneidensis* MR-1 (on1)	2616 (61.1)	2624	2650 (58.7)	2675
	ha,pe,se,wo	2665	2665	2665	2665
	ha,pe,se,wo,on1	2310	2310	2310	2310
	All 17 *Shewanella* strains (core CDS, CDS^c^)	1814 (42.4)	1814	1814 (40.2)	1814
Number of CDS in CDS^nc^ and subsets	Total CDS^nc^	2464	2427	2698	3066
	CDS^4^	851	851	851	851
	CDS^2-hp^	923	923	NA	NA
	CDS^2-sw^	NA	NA	482	482
	CDS^1-ha^	690	NA	NA	NA
	CDS^1-se^	NA	NA	1365	NA

Note: CDS^nc^, CDS not conserved in all *Shewanella*; CDS^4^, CDS^nc^ conserved in ha,pe,se and wo; CDS^2-hp^, non-CDS^4^ part of ha and pe orthologs in CDS^nc^; CDS^2-sw^, non-CDS^4^ part of se and wo orthologs in CDS^nc^; CDS^1-ha^, non-CDS^4^ and non-CDS^2^ part of ha CDS^nc^; CDS^1-se^, non-CDS^4^ and non-CDS^2^ part of se CDS^nc^; NA, not applicable.

**Figure 4:**
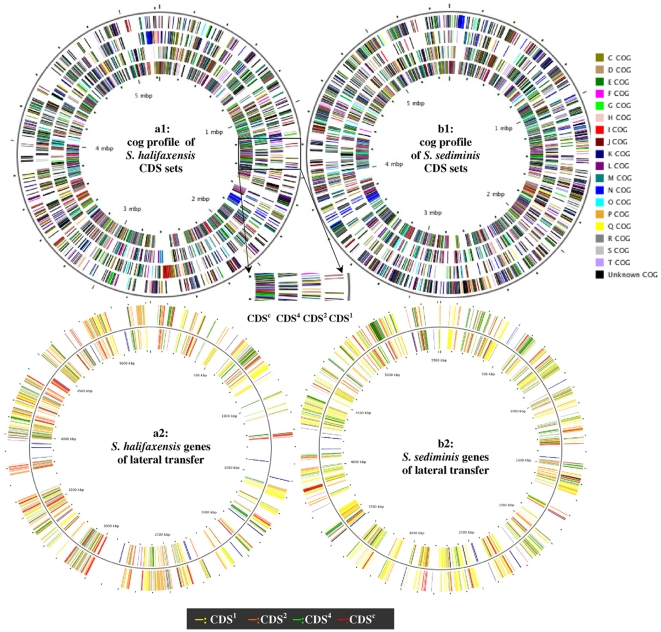
Circular maps of *S.halifaxensis* and *S. sediminis* genes in CDS subsets and laterally transferred. a1 or b1: cog profiles of CDS sets. CDS^c^, CDS^4^, CDS^2^ and CDS^1^ sets are explained in Table 2. One-letter abbreviations of cog are listed on the right side of b1. The corresponding color represent the following functional categories [69]: C, energy production and conversion; D, cell cycle control and mitosis; E, amino acid metabolism and transport; F, nucleotide metabolism and transport; G, carbohydrate metabolism and transport; H, coenzyme metabolism; I, lipid metabolism; J, translation; K, transcription; L, replication and repair; M, cell wall/membrane/envelope biogenesis; N, cell motility; O, post-translational modification, protein turnover, chaperone functions; P, inorganic ion transport and metabolism; Q, secondary metabolites biosynthesis, transport and catabolism; R, general functional prediction only; S, function unknown; T, signal transduction. a2,b2: Circular map of genes of lateral transfer. Red, green, brown or yellow color marks the genes in CDS^c^, CDS^4^, CDS^2^, or CDS^1^, respectively.

As shown in the phylogenetic tree prepared using the complete sequences of 16S rDNA (Fig. 2), *Shewanella* spp. were closely related to marine/aquatic γ-proteobacteria *Aeromonas*, *Vibrio*, *Photobacterium*, *Pseudoalteromonas*, *Colwellia,* and *Psychromonas*. To determine the *S. halifaxensis* and *S. sediminis* genes conserved in both *Shewanella* and these related bacteria, reciprocal best hit analysis (with a cut off E-value of -20) was conducted to compare all their deduced proteins with 15 other strains of *Shewanella* listed in Table 1. *S. halifaxensis* and *S. sediminis* genomes were found to share 1814 protein coding sequences (CDS) (42% total CDSs of *S. halifaxensis* and 40% total CDSs of *S. sediminis*) (Table 2) with all *Shewanella* compared, referred to as core *Shewanella* CDSs (CDS^c^, Fig. 4a1, b1). CDS^c^ were found highly conserved in the above related γ-proteobacteria, with more in the deep sea psychrophiles including *C. psychrerythraea* [20] (80%) and *P. profundum* [21] (79%), and slightly less in *Pseudoalteromonas* spp [22]–[24] (78%), *Aeromonas* spp. (78%) and *Vibrio* spp. (76%). Only 66–68% CDS^c^ were conserved in non-marine members of γ-proteobacteria including intestinal coliform *Serratia* (68%) and soil bacteria *Pseudomonas* (66%) living in warmer environments as compared to marine environment. This indicates that the *Shewanella* CDS^c^ are vertically inherited from a common γ-proteobacteria ancestor, with closer ties to γ-proteobacterial species adapted to the cold deep sea.

**Figure 5:**
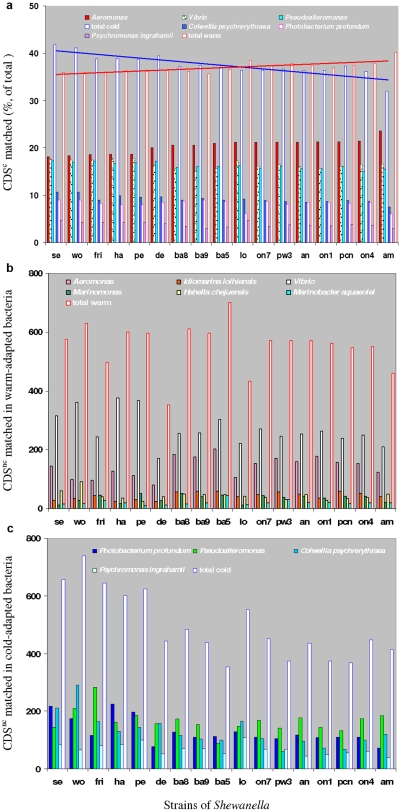
Major bacteria with proteins best matched to *Shewanella.* a, bacteria with top-hits matching proteins coded by CDS^c^; b, warm-adapted bacteria with top hits matching proteins coded by CDS^nc^; c, cold-adapted bacteria with top hits matching proteins coded by CDS^nc^. Bars represent bacteria with top hits matching CDS in strains of *Shewanella* as indicated on the x-axis (abbreviation listed in Table 1). The bar height indicates the count of hits in the bar-represented bacteria matching CDS^nc^ (b, c) or the percentage of hits relative to the 1814 CDS^c^ (a). The total hits in warm- or cold-adapted bacteria are indicated by the non-filled red or blue bars, respectively.

As shown in Fig 5a, the top-matches to CDS^c^ of all strains of *Shewanella* were mainly distributed in six genera including two mesophilic genera (*Aeromonas* and *Vibrio*) and four cold-adapted genera (*Photobacterium, Colwellia, Pseudoalteromonas* [*P. haloplanktis*, *P. atlantica*], *Psychromonas*). Among all strains of *Shewanella*, the five cold-adapted (*S. halifaxensis*, *S. sediminis*, *S. pealeana*, *S. woodyi* and *S. frigidimarina* [25]) had less CDS^c^ best matched in mesophilic *Aeromonas* but more best matched in the above-mentioned four cold adapted genera (>38% in each genera) and *Vibrio* (*V. fischeri* ES114> *V. parahaemolyticus* RIMD 2210633> *V. harveyi* ATCC BAA-1116> *V. vulnificus*∼*V.cholerae*) (Fig 5a). Interestingly, *Shewanella amazonensis* isolated from the tropical Amazon delta [26] and *Shewanella loihica* [27] isolated from an active Hawaii sea vent, which were known to tolerate >42°C, had the least CDS^c^ best matched in *P. profundum* SS9. Among all strains of *Shewanella*, *S. amazonensis* capable of tolerating 45°C also had the least CDS^c^ best matched in *C. psychrerythraea* 34H but the most CDS^c^ best matched in *Aeromonas* sp. (*A. hydrophila* ATCC7966 and *A. salmonicida* subsp. salmonicida A449). This observation suggests that *S. halifaxensis* and *S. sediminis* have a unique evolutionary history for adaptation to colder marine environments in contrast to the evolutionary history of *Shewanella* especially *S. amazonensis* and *S. loihica* adapted to warmer parts of marine/coastal environments.

Of the *Shewanella* CDS^c^, 333 were found to be absent in coliform and pseudomonads living in non-marine environments. Half of the 333 non-marine CDS^c^ were functionally uncharacterized and likely novel proteins. Among other half of 333 marine CDS^c^ functionally characterized, some were annotated as membrane proteins related to NaCl-tolerance and biofilm-formation. These included a Na^+^/H^+^ exchanger and a GTP-binding protein (Table 3) conserved in all closely related marine γ-proteobacteria including *Vibrio, Aeromonas, Colwellia, Pseudoalteromonas, Psychromonas,* and *Photobacterium*. A few other halotolerance-related proteins such as a Na^+^/H^+^ antiporter NhaC as well as a chloride channel, were conserved only in some of the above-mentioned marine γ-proteobacteria. Presence of these halotolerance genes in all *Shewanella* spp is consistent with their ability to tolerate high content of NaCl [9].

**Table 3:** *S. halifaxensis* and *S. sediminis* proteins for marine adaptation.

Proteins predicted		RefSeq protein accession^§^	Presence in^¥^							
			CDS^c^	CDS^4^	CDS^2-hp^	CDS^2-sw^	CDS^1-se^	O-shew	ma	n-ma
General halotolerance	GTP-binding signal proteins	YP_001673987	+						+	–
	divalent ions tolerance	YP_001672905	+						+/–	–
	chloride channel	YP_001673262	+						+/–	–
	TatB subunit, twin-Arginine translocation	YP_001676082	+						+/-	–
Na^+^/solute symporter	solute	YP_001674780		+				+		
	glutamate	YP_001675245		+				+		
		YP_001675528, 6399			+			+		
		YP_001476116,				+		+		
	neurotransmitter	YP_001675407, 6136		+				–		
	Eexcitatory amino acid	YP_001473092					+	+		
	proline	YP_001674892	+							
		YP001475470					+	+		
	dicarboxylate	YP_001673007, 3467, 3772, 3827	+							
		YP_001673866, 2587		+				+		
		YP_001673465		+				+		
		YP_001673061, 3630, 5246			+			+		
		YP_001472601					+	+		
	pantothenate	YP_001674802		+				–		
	multiple solutes	YP_001672900		+				–		
	nucleosides, 2	YP_001672959, 5694			+			+		
	SSS superfamily solute	YP_001473556					+	–		
	sulfate	YP_001472104					+	–		
Na^+^/H^+^antiporter or exchanger	antiporter NhaB	YP_001674065	+							
	antiporter NhaC	YP_001672969, 3907	+						+/–	–
		YP_001675151		+				+		
		YP_001672781; 5564		+				–		
		YP_001672313			+			–		
		YP_001675539, 5711, 6176			+			+		
		YP_001472418, 2875,5922				+		+		
	antiporter NhaA	YP_001673299			+			+		
	antiporter MnhD	YP_001473138, 5395					+	–		
	exchanger	YP_001672903, 6474	+							
		YP_001674101		+				+		
		YP_001673334			+			+		
		YP_001672941			+			–		
		YP_001475696, 5682					+	–		
Cation antiporter		YP_001675561			+			–		
		YP_001473129, 3132					+	–		
	Na^+^/Ca^++^, CaCA	YP_001672620, 5898		+				+		
	Heavy metal pump									
	heavy metal efflux pump	YP_001672513-2514; 2836-2837	+							
		YP_001676103		+				+		
	heavy metal	YP_001674420.	+							
	copper	YP_001675075.1	+							
Betaine/choline transporters		YP_001675036		+				+(–on1)		
		YP_001676241-6243		+				+(–on1)		
		YP_001672424, 2644, 2455, 5524,5960			+			+(–on1)		
		YP_001672644			+					
		YP_001474961					+	+(–on1)		
		YP_001473512					+			

Note: §: last four digits of accession number given; ¥, CDS sets listed in Table 2; o-shew, other *Shewanella*; ma, marine; n-ma, non-marine; +, present; -, absent; on1, *S. oneidensis* MR-1.

More than 60% of total CDSs of *S. halifaxensis* and *S. sediminis* were not in CDS^c^, and were designated as non-core part of CDS (CDS^nc^). Based on deduced protein sequence similarity, about 58–80% of CDS^nc^ had orthologs in cluster I *Shewanella*, and only <52% had orthologs in cluster II *Shewanella.* Therefore, the two RDX-degrading marine strains share more genes with obligate marine *Shewanella* in cluster I . Unlike CDS^c^, their CDS^nc^ were less frequent (39–28.7% in each bacterium) in finding orthologs (based on protein sequence blasting) in the genome of *Photobacterium*, *Vibrio, Colwellia,* or *Aeromonas*. However, the top hits to CDS^nc^ were still mainly distributed among the above four genera and *Pseudoalteromonas* (Fig 5b, c). More infrequent top hits occurred in *Psychromonas, Hahella, Idiomarina, Marinomonas* or *Marinobacter* (Fig. 5b,c). These results suggest that many of the CDS^nc^ likely originate via horizontal transfer from the marine gene pool and possibly account for their adaptation to deep sea environments.

Compared to other *Shewanella*, the cold-adapted obligate marine *S. halifaxensis, S. sediminis*, *S. pealeana* and *S. woodyi*, showed greater homology in CDS^nc^ to psychrophilic *P. profundum* strain SS9. Among all *Shewanella, S. sediminis* and *S. woodyi* also had more CDS^nc^ best matched in psychrophilic *C. psychrerythraea* strain 34H. The cold-adapted *S. frigidimarina* in cluster II had a greater identity in CDS^nc^ to cold adapted *Pseudoalteromonas*. In total, for any of the above five cold-adapted *Shewanella,* the number of CDS^nc^ best matched in cold-adapted non-*Shewanella* bacteria (Fig 5c) were higher than the number of CDS^nc^ best matched in warm-adapted bacteria (Fig 5b). In contrast, for any of mesophilic *Shewanella* mostly in cluster II, the number of CDS^nc^ best matched in mesophilic bacteria (Fig 5b) was higher than those best matched in cold-adapted bacteria (Fig 5c). This suggests that over the course of evolution, the five cold-adapted *Shewanella* have frequently exchanged their genes with bacteria living in cold environment.

To determine the genes unique to the two marine *Shewanella*, *S. halifaxensis* and *S. sediminis*, their CDS^nc^ were further separated into three non-overlapping subsets (Table 2). The first part was CDS^4^ shared among the four cold-adapted *Shewanella*: *S. halifaxensis*, *S. pealeana, S. woodyi*, and *S. sediminis*. The second part was grouped in CDS^2^, which was CDS^4^-excluded *S. halifaxensis* CDS^nc^ shared with *S. pealeana* (CDS^2-hp^) or *S. sediminis* CDS^nc^ shared with *S. woodyi* (CDS^2-sw^). The remaining non-CDS^4^ and non-CDS^2^ part of CDS^nc^ was referred to as CDS^1-ha^ for *S. halifaxensis* or CDS^1-se^ for *S. sediminis*. Blast analyses using protein sequence showed that all three subsets of CDS^nc^ of *S. halifaxensis* and *S. sediminis* were best matched in the cold deep sea bacterium *P. profundum*, with the exception of *S. sediminis* CDS^2-sw^ that was best matched in cold-adapted marine bacterium *C. psychrerythraea*. This indicates the influence of the two cold-adapted marine bacteria *P. profundum* and *C. psychrerythraea* in shaping the genomes of *S. halifaxensis* and *S. sediminis*.

Many *S. halifaxensis* and *S. sediminis* CDSs in CDS^4^, CDS^2^ and CDS^1^ (with some in CDS^c^) were opportunistic genes, not commonly conserved (or best matched) in closely related bacteria. These CDSs were identified, and presented in their circular chromosomal maps (Figure 4a2, 4b2). The annotated functions of these genes are available online from the Scalable Vector Graphics (SVG) Figures (Fig. S1 for *S. halifaxensis*, Fig. S2 for *S. sediminis*, supplementary materials). These genes were concentrated in 7–15 major genomic regions or islands on the chromosomes. Many integrase or transposase genes responsible for genomic recombination and rearrangement were found in the midst or near the ends of these islands. This evidence supports that these CDSs are genes likely horizontally transferred from bacteria in the same environment [28]–[30]. The majority of predicted foreign sources were proteobacteria, mainly in γ-division, with minor contributions from proteobacteria in α-, β-, δ- and ε-divisions, and most of them lived in marine and aquatic environment. Surprisingly, some were even members of gram-positive, spore-forming Firmicute, or photosynthetic bacteria such as *Cyanobacteria*. Close to 100 of them were freshwater bacteria including 49 terrestrial *Pseudomonas*, suggesting a history of close contact and relationship of *Pseudomonas* with the two marine strains of *Shewanella* over the course of genomic evolution.

### Genes for an Obligate Marine Lifestyle

Compared to other strains of *Shewanella*, obligate marine *S. halifaxensis* and *S. sediminis* were predicted to have more and unique CDSs associated with adaptation to sea salts and toxic chemicals (Table 3). The two strains had two common Na^+^-dependent nutrient symporters [31] found in CDS^c^ of all *Shewanella*. Unlike most non-obligate marine *Shewanella*, the two obligate marine strains had many unique Na^+^-dependent nutrient symporters found in their CDS^nc^ subsets. For example, both strains had eight Na^+^-dependent nutrient symporters in CDS^4^ set; and these were shared with obligate marine relatives *S. pealeana* and *S. woodyi*. Seven more Na^+^/nutrient symporters were found in CDS^2-hp^ of *S. halifaxensis* for transport of dicarboxylate, glutamate [Glu] and nucleosides. Three more Na^+^/nutrient symporters were found in CDS^1-se^ of *S. sediminis* for transport of Glu, proline [Pro], dicarboxylate and amino acids. Based on genome annotations of all *Shewanella*, Glu was predicted to be an essential precursor for biosynthesis of heme, nucleobase (purine, pyrimidine), Pro, peptiglycan, aminosugar, and fatty acids in *Shewanella*. Glu could also be a precursor to biosynthesis of aspartate (Asp) and lysine (Lys). Glu and Pro were also known osmoprotectants important for bacterial adaptation to salty environments [32]–[34]. Dicarboxylate and Glu metabolite 2-oxoglurate were part of the Krebs cycle (tricarboxylic acid, TCA) that supplies NADH and building blocks to biosynthesis of lipid, polysaccharides, proteins and nucleic acids. Requirement of Na^+^ as a motive force for transport of these essential growth substrates are consistent with the nature of *S. halifaxensis* and *S. sediminis* being obligate marine bacteria. Detection of many Na^+^-dependent nutrient symporters in *S. halifaxensis* and *S. sediminis* CDS^nc^ strongly demonstrates that these two marine species of *Shewanella* have evolved significantly for optimal adaptation to marine environment.

On the other hand, to maintain the difference in Na^+^ concentration across the membrane, marine bacteria have to remove Na^+^ leaked from sea water into cells [31]. Compared to other *Shewanella*, the four obligate marine *Shewanella* were found to have more unique Na^+^/H^+^ antiporter and/or exchanger genes[35], [36] (Table 3): 4 in CDS^4^, 7 in CDS^2-hp^ of *S. halifaxensis*, 3 in CDS^2-sw^ of *S. sediminis* and 4 in CDS^1-se^ of *S. sediminis*. The obligate marine *Shewanella* CDS^4^ also included genes for a Na^+^-translocating oxaloacetate decarboxylase [37] (YP_001673465) (Table 3) and two NADH-dependent Na^+^-pumps (Table 4) absent in genomes of many other *Shewanella*. The first Na^+^-pump (pump A) was a NADH:ubiquinone oxidoreductase ABCDEF system [38] (YP_001673869-73874, Table 4); the second pump (pump B) was composed of three unique NADH dehydrogenases (quinone) (YP_001675561-5569), a Na^+^/H^+^ antiporter, a cation antiporter and a multiple resistance and pH regulation protein F. The Na^+^-pump B system was also found (with homologs) in cold-adapted *S. frigidimarina* but rarely in other *Shewanella*. The three unique NADH dehydrogenases of Na^+^-pump B were most similar to those in α-proteobacteria *Parvibaculum lavamentivorans* DS-1 [39]. The Na^+^/H^+^ antiporter of pump B was closely related to the one in anaerobic mud-dwelling, anoxygenic phototrophic α-proteobacteria *Rhodospirillum rubrum* ATCC 11170 [40]. These results suggest that these marine *Shewanella* genomes have recruited the above transporter genes by horizontal gene transfer for better adaptation to marine environment.

**Table 4:** *S. halifaxensis* and *S. sediminis* proteins involved in electron transfer and biological reduction.

Protein predicted		RefSeq Protein Accession^§^	Presence in CDS subsets^¥^					
			CDS^c^	CDS^4^	CDS^2-hp^	CDS^1-ha^	CDS^2-sw^	CDS^1-se^
NADH oxidoreductase	NADH: quinone oxidoreductase ABCDEF	YP_ 001675388-5392	+					
		YP_ 001673869-3874		+				
	NADH: quinone oxidoreductase 4L	YP_001675565		+				
	NADH dehydrogenase	YP_001675561-5569		+				
		YP_001675702, 5693			+			
		YP_001472992, 5459					+	
		YP_001473136						+
	NADH:flavin oxidoreductase (old yellow enzyme)	YP_001674250	+					
		YP_001474442		+				
		YP_001674320-4324			+			
		YP_001473404					+	
		YP_001474445, 4320						+
cytochrome *c*	biogenesis protein	YP_001676298, 6300	+					
	biogenesis system	YP_001672783		+				
	*c*1	YP_001675828, 6302, 6421	+					
	*c*2	YP_001673338	+					
		YP_001674623, 2784, 2785, 2801, 4688, 4997,5136		+				
		YP_001674990			+			
		YP_001673258, 3261, 6150			+			
		YP_001672848, 2850				+		
		YP_001476097					+	
		YP_001472093, 2254, 3044, 3141, 3269, 3886, 4660						+
	*c*3	YP_001474443						+
	*c*553	YP_001473683						+
	flavo-	YP_001672566, 3010,5057		+				
		YP_001676149, 6165, 5652, 6152			+			
		YP_001472038, 5916						+
	flavo- flavin subunits	YP_001472085, 5910						+
	tetraheme	YP_001672567		+				
		YP_001675654, 6166			+			
		YP_001472083, 5917						+
	decaheme	YP_001674993, 4996, 5867		+				
		YP_001473265					+	
		YP_00147 2737, 2102, 3266, 3267						+
	decaheme, MtrF	YP_001674992			+			
nitroreductase		YP_001674672	+					
		YP_001673967		+				
		YP_001673048, 3510			+			
		YP_001474717						+
nitrite reductase	cytochrome, ammonia−forming	YP_001673490, 5126			+			
		YP_001675128, 3320				+		
	(NAD(P)H) small, large subunit	YP_001474499, 4500					+	
	cytochrome, ammonia−forming	YP_001472427, 5385, 5476						+
	formate−dependent, nrfD protein	YP_001472257						+
Nitrate reductase	periplasmic, large subunit	YP_001672949		+				
	NapC/NirT cytochrome c domain	YP_001672946		+				
	NapB, cytochrome c subunit	YP_001672519		+				
		YP_001675476-5477			+			
		YP_001672411-2415				+		
		YP_001473690-3694					+	
		YP_001474534					+	
	cytochrome *c* subunit	YP_001473653, 3654					+	
	periplasmic, NapE	YP_001475090						+
	ntrate/TMAO reductase	YP_001473684						+
TMAO reductase	TorA, TorT	YP_001675621-5625		+				+
	cytochrome *c*, TorC	YP_001675619	+					
DMSO reductase		YP_001675134, 5133		+				
		YP_001672640				+		
	DmsA/YnfE family A subunit	YP_001472091, 2100, 3046, 3143, 4658,						+
	subunit B	YP_001472099						+

Note: §, last four digits of accession number given; ¥, CDS sets listed in Table 2; +, present.

To adapt to the high osmotic pressure of seawater, *S. halifaxensis* and *S. sediminis* were also found to have the genes responsible for transport of osmoprotectants betaine/choline/carnitine (BCCT) [32] (Table 3). One BCCT transporter gene was found in genomes of most *Shewanella* except the freshwater strain *S. oneidensis*. The CDS^4^ of the four obligate marine *Shewanella* had two transporter genes for BCCT and one ABC-type transporter gene for glycine betaine/L−proline also known as osmoprotectants (Table 3). In addition, *S. halifaxensis* (three in CDS^2-hp^) and *S. sediminis* (two in CDS^1-se^) also had some unique BCCT transporter genes for better osmotic pressure protection (Table 3).

Marine sediment is known to be a final destination of many toxic chemicals in the eco-system. *S. halifaxensis* and *S. sediminis* as two marine sediment bacteria were found to have abundant unique genes for acriflavin resistance and toxic compound extrusion (MATE, multidrug and toxic compound extrusion efflux). They had 14 toxin resistance genes found in CDS^c^ shared with all *Shewanella*. Twenty-five more toxin resistance and efflux genes were predicted in CDS^4^ set that were unique to all of the four obligate marine *Shewanella*. More toxin-resistance genes were found in the specific CDS^nc^ subsets of *S. halifaxensis* (18 in CDS^2-hp^, 7 in CDS^1-ha^) and *S. sediminis* (6 in CDS^2-sw^, 26 in CDS^1-se^). Sixteen toxin-resistance genes of the two marine strains (3 in CDS^4^, 4 in CDS^2-hp^, 1 in CDS^2-sw^, 8 in CDS^1-sw^) were absent in most non-obligate marine *Shewanella*. Four pairs of the RND family MFP subunits and effluxes were absent in *S. oneidensis* MR-1 known to adapt to freshwater lakes, indicating their association to marine sediment. Furthermore, the four obligate marine strains had several effluxes for pumping out heavy metals, calcium and potassium which were not found in genomes of many other *Shewanella* (Table 3).

The present genomic comparison between obligate marine and non-obligate marine species of *Shewanella* clearly shows that marine species have evolved to contain more unique genes responsible for resistance to toxins and high osmotic pressure. Most importantly the present comparison for the first time demonstrates that obligate marine species have evolved to take advantage of the constantly high content of Na^+^ in marine environment as a driving force for uptake of essential nutrients.

### Amino Acid Composition Profiles of Cold-Adapted γ-Proteobacteria

Thermophilic proteins adapted to higher temperatures were known to have stable structures characterized by having certain amino acid residues substituted by Pro [41], [42] and Arginine (Arg) [43]. Proteins in psychrophiles were believed to have looser structures and increased conformation flexibility to allow for higher specific activity at low temperatures [44]–[46]. An earlier study showed replacement of Arg by Lysine (Lys) or Serine (Ser), Valine (Val) by Alanine (Ala) or Isoleucine (Ile), Lys by Ser or Asparagine (Asn), and Glu by Ala, in 21 psychrophilic bacterial enzymes as compared to their meso/thermophilic homologs [47].

**Figure 6:**
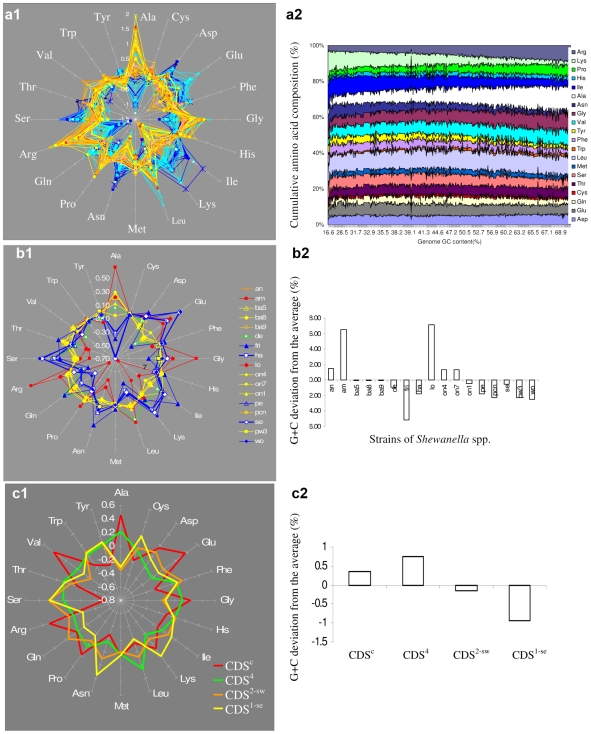
The characteristic profiles of amino acid composition and G+C content in cold-adapted γ-proteobacteria. Bacterial abbreviations for b1, b2 are listed in Table 1. In general in a1,b1,c1, each axis represents the deviation of one amino acid composition in one bacterium (or CDS set) from the average in all bacteria compared. One line shows the amino acid deviation in one specific bacterium or CDS set. a1, deviation in one bacterium from the average of all 118 proteobacteria as given in Fig 2; b1, deviation in one strain of *Shewanella* from the average of 17 strains of *Shewanella*; c1, deviation in one set of CDS from the average of total CDSs in *S. sediminis*. Warm-adapted bacteria were represented by yellow, orange and red lines and symbols; cold-adapted bacteria were represented by the blue lines and symbols. a2, correlation between amino acid compositions of all 623 sequenced bacteria and their G+C content. Bacteria are represented by their G+C content. Amino acid compositions are displayed in a cumulative format. b2, deviation of G+C content in one strain of *Shewanella* from the average of all 17 strains of *Shewanella*; c2, deviation of G+C content of a set of CDS from the value of total CDS in *S. sediminis*. In a1, b1, blue curve indicates the psychrophilic (or psychrotrophic) bacteria; yellow, orange, and green curves indicate bacteria living in warm environments.

To provide insight into how bacterial proteins evolve to adapt to cold environment, *S. halifaxensis, S. sediminis* and three other cold-adapted *Shewanella* were compared on proteome amino acid composition with 12 other mesophilic strains of *Shewanella* as well as 93 other γ-proteobacteria living in either warm or cold environments (listed in Fig 2). As shown in Figure 6a1, among the total 110 γ-proteobacteria (including 17 strains of *Shewanella*) compared, the marine/aquatic γ-proteobacteria living in generally colder environments (marked by the blue color, Fig 2) contrasted to those living in warmer environments (marked by the yellow or orange color, Fig 2) with regards to composition of certain amino acids. The typical warm-adapted bacteria were soil pseudomonads and animal intestinal coliforms known to grow optimally at 30–37°C. Bacteria living in colder environment were found to be lower in the contents of Ala, Pro, Arg, Glycine (Gly) and Leucine (Leu), but higher in contents of Asp, Asn, Ile, Lys and Ser.

Interestingly, among the 110 strains compared (Fig 6a1), the amino acid composition profiles of cold-adapted *S. sediminis, S. woodyi, S. halifaxensis, S. pealeana* and *S. frigidimarina* were closer to psychrophilic species of other genera than to mesophilic strains of *Shewanella* in cluster II. This suggests a temperature-driven protein divergent evolution pathway within the same phylogenetic group. This trend was clearly observed among *Shewanella* and 6 other closely related marine γ-proteobacterial genera (31 strains). The 10 cold adapted bacteria included two strains of *Pseudoalteromonas*, 5 strains of *Shewanella*, and one strain of *Colwellia*, *Photobacterium*, or *Psychromonas* (dark blue triangles, Fig 2). The 21 warm-adapted bacteria included 12 strains of *Shewanella*, 2 strains of *Aeromonas* and 7 strains of *Vibrio*. Compared to the warm-adapted marine γ-proteobacteria, the cold-adapted were lower in the contents of Ala, Arg, Pro, Leu, Trp and His, but higher in the contents of Ile, Lys, Asp, Asn, Ser and Tyr. As shown in Table 5, the above differences in total proteins between the two groups of marine γ-proteobacteria were significant because the p-values were less than 0.01.

**Table 5:** Amino acid composition and G+C content comparison between cold- and warm-adapted γ-proteobacteria.

Nucleotide or amino acid (%)	Δ (cold-warm)		marine γ-proteobacteria						*Shewanella*					
	From GC correlation^§^	By proteins	Total proteins			Common proteins			total proteins			common proteins		
			cold	warm	p	cold	warm	p	cold	warm	p	cold	warm	p
G+C	–	–	42.9	48.3	0.00	43.8	49.2	0.00	44.6	48.0	0.01	45.2	48.6	0.01
A+G	+	+	52.1	51.4	0.00	52.3	51.6	0.00	51.9	51.4	0.00	52.0	51.3	0.00
Ile	+	+	6.8	6.0	0.00	6.6	6.0	0.00	6.5	6.1	0.00	6.4	6.1	0.00
lys	+	+	5.8	5.3	0.01	5.7	5.3	0.00	5.5	5.2	0.00	5.5	5.2	0.00
Asp	nc	v	5.3	5.2	0.01	6.1	6.2	0.76	5.4	5.2	0.00	5.6	5.5	0.06
Asn	+	+	4.5	3.9	0.00	4.1	3.6	0.00	4.2	3.8	0.00	3.9	3.7	0.00
Ser	nc	+	6.8	6.4	0.00	6.1	5.7	0.01	6.9	6.4	0.00	6.3	6.0	0.08
Tyr	+	v	3.1	2.9	0.00	2.7	2.7	0.34	3.0	2.9	0.03	2.7	2.7	0.59
Ala	–	–	8.5	9.2	0.00	8.9	9.6	0.00	8.7	9.5	0.00	9.0	9.7	0.00
Leu	nc	–	10.6	11.0	0.00	10.2	10.5	0.13	10.6	11.1	0.00	10.5	10.9	0.04
Pro	–	–	3.7	4.0	0.00	3.9	4.1	0.00	3.8	4.0	0.00	3.9	4.1	0.00
Arg	–	–	4.2	4.8	0.00	4.7	5.1	0.00	4.3	4.7	0.00	4.7	4.9	0.14
Trp	nc	–	1.2	1.3	0.00	1.0	1.1	0.01	1.2	1.3	0.00	1.0	1.1	0.13
His	nc	–	2.3	2.4	0.01	2.2	2.3	0.11	2.3	2.3	0.09	2.2	2.3	0.31

Note: Cold adapted marine γ-proteobacteria included five cold-adapted *Shewanella* (dark blue triangles, Fig 2) and two strains of *Pseudoalteromonas,*one strain of *Colwellia, Photobacterium* or *Psychromonas* indicated (dark blue circles, Fig. 2). Warm-adapted marine γ-proteobacteria included the rest 12 strains of *Shewanella*, 2 strains of *Aeromonas* and 7 strains of *Vibrio* (Light blue colour, Fig 2). Note: Δ (cold-warm), difference between cold and warm adapted; cold, cold adapted bacteria; warm, warm-adapted bacteria; §, amino acid composition was predicted by G+C correlation shown in Fig 6a2; nc, no correlation with GC;+, positive value; –, negative value; v, vary; p, p-value.

This trend was also true for homologous proteins among the above 31 marine γ-proteobacteria. The p-values on differences in compositions of Ile, Lys Asn, Ser, Ala, Pro, Arg and Trp were lower than 0.01. The lower Leu (p = 0.13) and His (p = 0.11) contents as well as the higher Asp content (p = 0.76) were also observed in cold-adapted marine γ-proteobacteria, but the differences appeared to be insignificant because the p-values were relatively high (>0.05, Table 5).

Furthermore, a difference on composition of above mentioned amino acids was observed between the 5 strains of *Shewanella* that did not tolerate 30°C and the 12 mesophilic strains of *Shewanella* that tolerated 30°C. As shown in Table 5, these differences in total proteins were significant with p-values below 0.01 for most of above mentioned amino acids except His (p = 0.09) (Table 5). Among the homologous proteins of the 17 strains of *Shewanella* (coded by CDS^c^), the five cold-adapted *Shewanella* were also clearly lower in Ala, Leu, Pro, and Arg contents, but higher in Ile, Lys, Asn, and Ser contents than the warm-adapted *Shewanella* (Fig 6b1). The differences on the above amino acids were significant with p values below 0.04 (Table 5). The two most warm-adapted *Shewanella*, *S. amazonensis* and *S. loihica*, which could grow at temperature >42°C, were especially higher in the content of Ala, Leu, Pro, or Arg than the rest *Shewanella*. The present study shows for the first time that the proteomes of cold-adapted γ-proteobacteria have lower contents of Ala, Arg and Pro as well as higher contents of Ile, Lys and Asn, as compared to closely related γ-proteobacteria adapted to warmer environment. This suggests that this characteristic amino acid profile observed in cold-adapted γ-proteobacteria (or subsets) might be beneficial to protein conformation flexibility of total proteome especially of those proteins produced and active under low temperature conditions [22], [43], [44], [46]–[47].

Some of the composition differences observed in certain amino acids between warm-adapted and cold-adapted bacteria could be attributed to the differences in the GC content of their genomes. As shown in Figure 6a2, among the 623 sequenced bacteria, Arg, Pro, Ala and Gly contents were proportional to their genome GC contents, that is, the higher the GC content was, the higher the content of the four above-mentioned amino acids were. The opposite was also true for Ile, Lys, Asn, or Tyr. As shown in Table 5, the cold-adapted marine γ-proteobacteria were indeed lower in GC content than mesophilic ones with a p-value <0.001. Among the 17 strains of *Shewanella*, the five strains that did not tolerate 30°C, were significantly lower in GC content for both total CDS and CDS^c^ than strains of *Shewanella* that tolerated 30°C (Table 1), with p-value <0.01. As shown in Figure 6b2, *S. amazonensis* and *S. loihica* that tolerated >42°C were especially higher in G+C content than the rest of *Shewanella*. Therefore the low content of Ala, Pro, Arg, and high content of Ile, Lys and Asn observed in cold-adapted bacteria are consistent with their low GC content as compared to warm-adapted bacteria.

As shown in Figure 62a, no correlation was observed between GC content and compositions of other amino acids including Asp, Ser, Leu, Trp and His. The latter amino acids also shifted their compositions in cold adapted bacteria as compared to warm-adapted bacteria especially for total proteins (Table 5, Fig 6). This suggests that bacteria may adjust their protein amino acid compositions to better adapt to changes in temperatures without necessarily affecting or involving changes in their genome GC contents.

For *S. sediminis* (Fig 6c1) or *S. halifaxensis* (data not shown), the proteins coded by CDS^1^ were also lower in Ala, Arg and Pro contents and higher in Ile, Asn, and Ser contents than those coded by CDS^c^. This trend is similar to those observed between cold-adapted and warm-adapted bacteria as shown in Fig 6a1 and 6b1. These results further suggest that the proteins coded by CDS^1^ of the two *Shewanella* are more cold-adapted than those coded by core *Shewanella* genes (CDS^c^), inherited from a common proteobacterial ancestor. The lower contents of Ala, Arg and Pro in proteins coded by CDS^1^ correlated well with the lower GC content of CDS^1^ in *S. halifaxensis* (42.8%) or in *S. sediminis* (45.8%) as compared to CDS^c^ (*S. halifaxensis*, 45.8%; *S. sediminis*, 47.0%) (Fig 6c2). As discussed in above sections, many CDS^1^ of *S. halifaxensis* and *S. sediminis* were laterally transferred (Fig 4a2 and b2), mostly from cold-adapted bacteria. These results further provide evidence for adaptation of *S. sediminis* and *S. halifaxensis* genomes to the cold sea by recruiting cold-adapted genes from bacteria living in a similar environment during later stage of genomic evolution.

### Genes and Proteins Involved in Anaerobic RDX Metabolic Pathways

**Figure 7:**
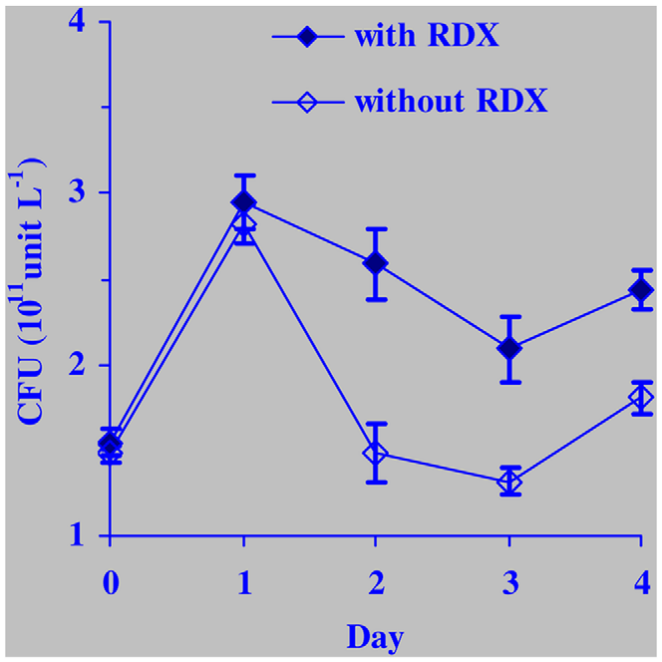
Anaerobic growth of *S.halifaxensis* HAW-EB4 on RDX as the sole terminal electron acceptor. CFU, colony-forming unit.

As two RDX-mineralizing anaerobic bacteria dominant at a UXO-contaminated marine sediment site, *S. halifaxensis* and *S. sediminis* were also capable of reducing several other explosives including percholorate (data not shown), dinitrotoluene and trinitrotoluenes (Table 1). For comparison, the three most closely related reference bacteria *S. pealeana, S. woodyi* and *Shewanella hanedai*, were tested for their ability to remove explosives. The results showed that the three reference bacteria not from the UXO-contaminated site, where *S. halifaxensis* and *S. sediminis* were dominant, displayed more than two times lower RDX-metabolic activity and little percholorate-removal activity under the same experimental conditions (Table 1). Presently, we also found that *S. halifaxensis* cells had a better viability when they were incubated in the MB-20 marine medium in the presence of a saturated amount of RDX as the sole terminal electron acceptor than in the absence of RDX (Fig. 7). A recent study using the molecular DGGE method also showed that anaerobic incubation with a nitrated compound, 2, 4-dinitrotoluene (DNT) that was reported to be present at the contaminated site, led to enrichment of *Shewanella* in marine sediment sampled from this Halifax UXO site [16]. These findings together demonstrate that *S. halifaxensis* and *S. sediminis* are well-adapted for remediation of explosives (RDX, DNT, TNT, and ClO_4_^−^) than most closely related reference strains of *Shewanella* not found at the contaminated site.

Remediation of the above explosives requires an initial reductive step involving reductases and electron donors. As shown in Table 4, *S. halifaxensis* and *S. sediminis* genomes indeed contained more and unique genes for electron-transfer and reductases than *S. pealeana* and *S. woodyi*. For example, *S. sediminis* had six more subunits of DMSO reductase (five A subunits and one B subunit) than *S. woodyi*.

#### *C*-type cytochrome and RDX denitration pathway

*C*-type cytochromes were electron-transferring proteins destined to enter the periplasmic space, and in some cases are secreted to the outer membrane by the Type II protein secretion system [48] for transferring electrons to extra cellular electron acceptors. Strains of *Shewanella* were known to use *c*-type cytochrome for periplasmic reduction of nitrate, fumarate [49], TMAO, and extra cellular reduction of DMSO [50] and metal oxides [48], [51]–[55]. Present genomic analysis showed that *S. sediminis* (48 reported in Genbank, 26 more identified in this study) and *S. halifaxensis* (36 reported in Genbank, 14 more identified in the present study) genomes had a higher number of *c*-type cytochrome genes compared to other strains of *Shewanella* (Table 4). Many were unique and novel *c*-type cytochromes shared by the two RDX degraders. They had several tetraheme cytochrome *c* genes absent in many other *Shewanella*. Two cytochrome *c* genes were absent in *S. woodyi* and *S. pealeana* (Table 4), but present in *S. oneidensis* that was also positive for RDX reduction (J. S. Zhao, D. Manno, J. Hawari, unpublished results). *S. halifaxensis* and *S. sediminis* also shared four additional pairs of flavo- and tetraheme- cytochrome *c* genes, one pair absent in any other *Shewanella*, three others present in the slower RDX-degrading *S. pealeana*, but not in the much poorer-RDX degrading *S. woodyi*. The latter three pairs of flavo- and tetraheme- cytochrome c were closely related to those in ε-proteobacteria *Wolinella succinogenes* DSM 1740 or *Campylobacter concisus* 13826. *S. sediminis* had 19 *c*-type cytochrome genes absent in closely related *S. woodyi* (Table 4) which displayed a five times lower RDX-metabolic activity (Table 1). Previously, cytochrome-like hemeprotein XplA in Actinomyces [56] and a cytochrome P450 in rabbit liver [57] were reported for aerobic RDX-reduction activity. The *xplA* was not found in either *S. halifaxensis* or *S. sediminis*. Interestingly, *c*-type cytochromes-abundant freshwater lake sediment bacteria *Geobacter* (97 in *Geobacter sulfurreducens* and 63 in *Geobacter metallireducens*) were also recently detected for their ability to reduce RDX [58].

**Figure 8:**
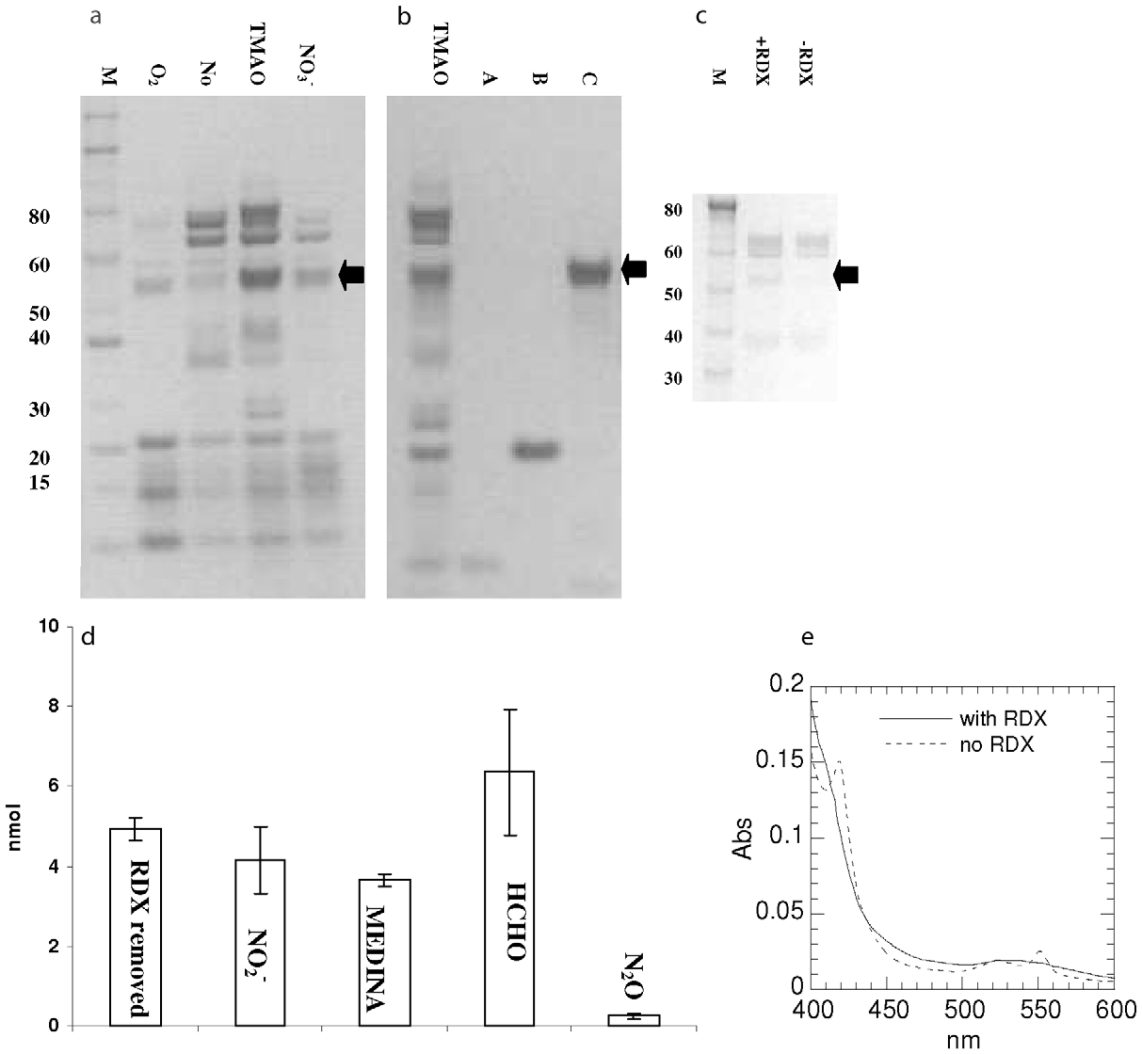
The 52 kDa cytochrome involved in RDX metabolism. a-c, heme-stained SDS-PAGE. a, induction by terminal electron acceptors (O_2_, nitrate, TMAO, none). c, induction by RDX (control, -RDX); b, isolated cytochrome in lane C. d, cytochrome-catalyzed RDX denitration and products. e, UV-visible spectra of the isolated cytochrome during incubation with NADH in the presence (—) or absence (---) of RDX.

**Figure 9:**
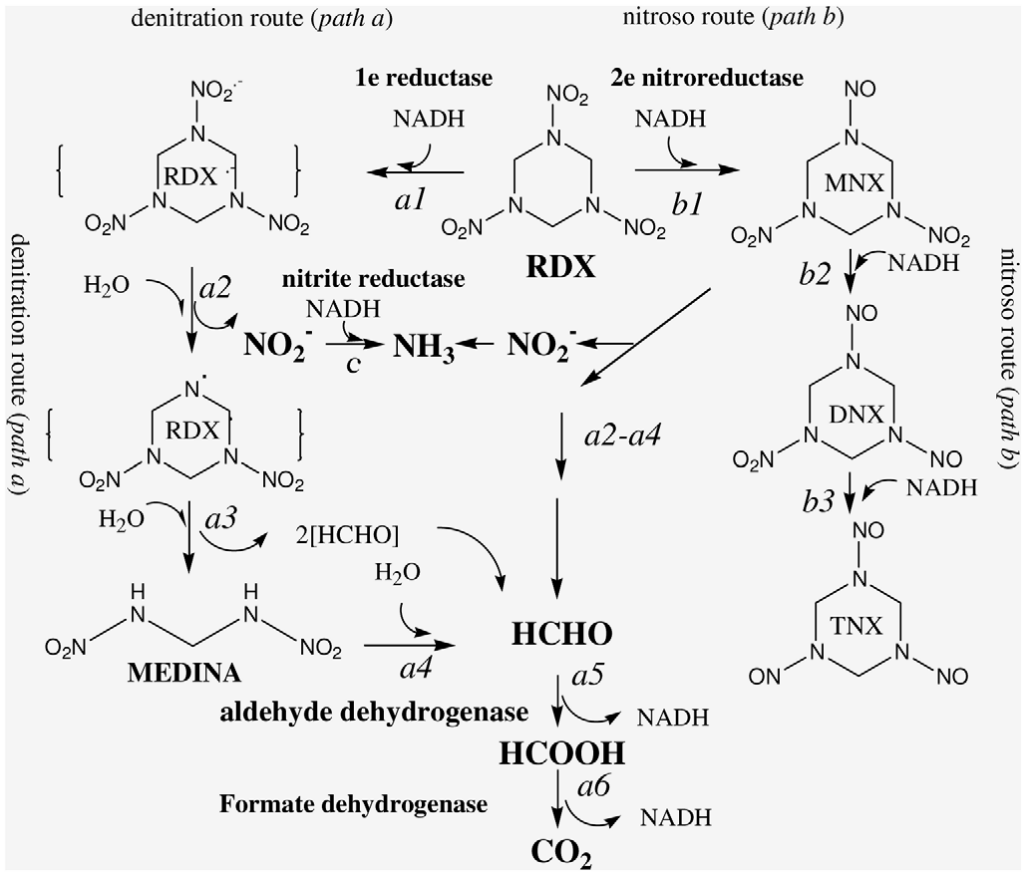
RDX metabolic pathways in *S.halifaxensis* and the enzymes involved. This figure was modified from the pathways published previously [57], [59].

In an earlier study, we reported that *S. halifaxensis* cells respiring on TMAO were optimal for removal of RDX, and the total *c*-type cytochromes were suggested to be involved in RDX reduction [59]. TMAO-grown cells were indeed found to contain higher contents of several *c*-type cytochromes than the slower RDX-degrading cells pre-grown on other electron acceptors (Fig. 8a). In this study, while we attempted to purify the RDX-reducing protein from TMAO-grown cells, a protein fraction containing a major cytochrome *c* (M = 52 kDa, indicated by an arrow in Fig. 8b) was found to display a NADH-dependent RDX-reducing activity (0.43 mg · L^−1^· h^−1^) (0.15 mg · ml^−1^ protein). Products analysis showed that RDX was degraded by a mono-denitration pathway to give MEDINA and HCHO (Fig. 8d) as observed for step a1-a2 in whole cells (Fig 9, a3-a4, abiotic reaction [57]). As shown in Figure 8e, the 420 and 552 nm peaks of the reduced form of the *c*-type cytochrome disappeared during RDX reduction, indicating RDX oxidation of cytochrome. Compared to cells incubated in the absence of any terminal electron acceptor, cells incubated with RDX as the sole terminal e-acceptor produced a higher content of the 52 kDa cytochrome (pointed by an arrow in Fig. 8c), indicating the capacity of RDX to up-regulate biosynthesis of this cytochrome. In the nitrate- and aerobic-grown cells that displayed a lower RDX-degradation activity, this cytochrome was only produced in smaller amounts (pointed by an arrow in Fig. 8a). This demonstrates that the 52 kDa cytochrome is involved in RDX denitration. It should be mentioned that other cytochromes, especially those low molecular weight cytochromes dominant in aerobic and nitrate grown cells, did not catalyze NADH-dependent RDX degradation. Using a proteomic approach, the 52 kDa cytochrome in the SDS-PAGE band was found to have a sequence best matching a multiheme C_552_ cytochrome (467 amino acids, YP_001673120, Shal_0886) in its genome. An orthologous cytochrome was also found in *S. sediminis* with a similarity of 86%.

Interestingly, in the present study, RDX degradation activity was also found in a cytochrome *c* isolated from *Saccharomyces cerevisiae* (Sigma C2436), removing 0.42 mg L^−1^ h^−1^ of RDX under the same protein concentration (0.11 mg ml^−1^ protein). This further demonstrates the capacity of certain *c*-type cytochrome to mediate electron transfer from NADH to RDX. This is also consistent with the observation in Figure 7, showing that RDX improved viability of *S. halifaxensis*. Since RDX was the sole terminal electron acceptor in the carbon and nitrogen-rich MB-20 medium, the better viability of cells is likely caused by a weak RDX-respiring process involving *c*-type cytochrome.

Nitrite was an intermediate of the RDX denitration metabolic pathway (Fig 9). As shown in Table 4, *S. sediminis* and *S. halifaxensis* genomes were found to contain genes coding nitrite reductases. The genomes of the two RDX-adapted *Shewanella* contained some nitrite reductase genes shared with other *Shewanella*. They also coded some unique nitrite reductases absent in their close relatives. For example, *S. sediminis* genome had three genes for ammonia-forming cytochrome nitrite reductases and one gene for nrfD protein of formate-dependent nitrite reductase absent in *S. woodyi*; *S. halifaxensis* had two genes for ammonia-forming cytochromes absent in *S. pealeana*. This observation suggests that the two RDX degraders have evolved to increase their genetic potential to convert the toxic compound RDX to ammonium, a nitrogen source for growth of many bacteria in the marine eco-system including *Shewanella*.

#### Nitroreductase and RDX nitroso pathway

*Shewanella* were also known to reduce RDX or DNT via a two-electron reduction of the nitro group to give nitroso derivatives (step b1-b3, Fig 9). Earlier studies described that a type I oxygen-insensitive 2e-transfer nitroreductase in coliform *Enterobacter cloacae* could slowly reduce RDX [60], [61]. Two genes for 2e-transfer nitroreductases were found in genomes of *S. sediminis* and *S. halifaxensis* genomes, and they all were conserved in reference strains *S. pealeana* and *S. woodyi*: one was coded by CDS^c^ and conserved in all *Shewanella*, another was coded by CDS^4^ and absent in many other *Shewanella*. *S. halifaxensis* CDS^2-hp^ coded two additional nitroreductases both shared with *S. pealeana*; one (YP_001673510, Table 4) was conserved in all coliforms (*Salmonella*, *Shigella, Escherichia, Enterobacter, Klebsiella*) but absent in all other *Shewanella*. *S. sediminis* had one unique nitroreductase gene (in CDS^1-se^, YP_001474717, Table 4) closely related to the one in *Methylibium petroleiphilum* PM1 but absent in *S. woodyi*. On the other hand, old Yellow family enzymes were also reported for their TNT-reducing activity [62]. *S. halifaxensis* and *S. sediminis* indeed had Old Yellow family enzymes (NADH: flavin oxidoreductases); several of them were unique to the two explosive degraders. Overall, since the 2e-nitroreductase was only previously reported for a poor RDX reducing activity, and these nitroreductases and Old Yellow family flavo enzymes were conserved in slow RDX-degrading strains of *Shewanella* as well as coliforms, they were unlikely to contribute significantly to RDX metabolism in *S. halifaxensis* and *S. sediminis*.

#### Aldehyde and formate dehydrogenases for RDX carbon mineralization

Aldehyde and formate dehydrogenase likely involved in oxidation of HCHO, a ring cleavage product of RDX, to CO_2_ (Fig. 9, step a5, a6) were also predicted in the genomes of *S. sediminis* and *S. halifaxensis*. *S. sediminis* (Ssed) and *S. halifaxensis* (Ssed) shared two aldehyde dehydrogenase genes, one located in a betaine metabolic operon (Ssed, YP_001472856- YP_001472860; Shal, YP_001673283-YP_001673287) and another in a proline metabolic operon (Shal, YP_001674771-YP_001674772; Ssed, YP_001473499-YP_001473500). The two RDX-degraders shared two formate dehydrogenase genes, one (Shal: YP_001672333-YP_001672346; Ssed: YP001476145.1-YP001476158.1) conserved in most *Shewanella* except *S. denitrificans,* and another (Ssed: YP_001475940-YP_001475948; Shal: YP_001676207- YP_001676223) absent in many *Shewanella* including *S. woodyi* and *S. pealeana*. The formate dehydrogenase operons were composed of genes for *c* and γ subunits related to *P. profundum*. Interestingly, *S. halifaxensis* had four more formate dehydrogenase systems than *S. sediminis*, consistent with its capacity to mineralize a higher percentage of RDX to CO_2_ [12]. One system was composed of α,β,γ subunits conserved in *S. pealeana* and coliform bacteria (YP_001672725- YP_001672735) such as *E. coli* ATCC 8739, *Enterobacter* sp. 638, and *Citrobacter koseri* ATCC BAA-895. Two other systems were composed of γ-subunits conserved in *P. profundum* SS9 and 4Fe-4S ferredoxin binding domain proteins (YP_001676214-YP_001676222) related to either *Desulfitobacterium hafniense* Y51 or *Aeromonas*. The fourth formate dehydrogenase gene (YP_001673633-YP_001673636) was associated with a Fe-only hydrogenase present in *Syntrophomonas wolfei* subsp. wolfei str. Goettingen. The present genomic analyses show that, as shown in Figure 9, the two *Shewanella* have evolved to recruit many genes for oxidization of [HCHO], a RDX ring cleavage product, to CO_2_, yielding NADH. The NADH generated should be sufficient for reduction of NO_2_^-^ to ammonia. Therefore the two strains of *Shewanella* are predicted to have the genomic potential to mineralize RDX and use it as an energy and nitrogen source for bacterial growth if other required co-factors are provided.

### Conclusions

In summary, the present study represents the first comprehensive genomic comparison of psychrophiles or psychrotrophs with closely related mesophiles within the same genus (*Shewanella*) or division (γ-proteobacteria). As a result we were able to discover that a shift in GC content and composition of certain amino acids in cold-adapted bacteria. As obligate marine bacteria, the two RDX degraders' genomes were found to be enriched with genes for halotolerance or for Na^+^-driven import of essential nutrients from the environment. Furthermore, the two strains of *Shewanella* had genes for *c*-type cytochromes, nitroreductases, and nitrite reductases for initial RDX reduction, as well as aldehyde and formate dehydrogenases for mineralization of RDX to CO_2_. These genomic evolution analyses and experimental data presented herein explained why *S. halifaxensis* and *S. sediminis* were dominant at UXO contaminated marine sediment sites. These comparative genomic and proteomic analyses represented the first attempt to understand how environmental bacteria were naturally selected in a specific contaminated site for survival and for *in-situ* remediation of pollutants, the explosive RDX in the present case.

## Materials and Methods

### Genome Sequencing and Annotation

Sequencing, assembly, finishing, pipeline annotation, and verification were completed by staff members of the US Department of Energy Joint Genome Institute (JGI, Walnut Creek, CA, USA) using standard protocols as published online (http://www.jgi.doe.gov/sequencing/protocols/prots_production.html). Briefly, the whole-genome shotgun sequencing method and the 3-Kb, 8-Kb, and 40-Kb DNA libraries were used for sequencing from both sides of library inserts. Sequences were aligned using genome assemblers to produce draft assembly, followed by gap closing, quality improvement, and assembly verification. Genome annotation was performed by Oak Ridge National Laboratory using automated annotation Genome Portal. The genomes were published in NCBI with accessions listed in Table 1.

### Comparative Genomics

In order to determine the complete genomic sequence similarity, and large scale genomic arrangement occurred over the course of evolution between *S. halifaxensis*, *S. sediminis* and 15 other species of *Shewanella*, whole-genome pair wise alignment was conducted using Progressive Mauve (Multiple alignment of conserved genomic sequence) 2.2.1 [63] (http://gel.ahabs.wisc.edu/mauve/mauve-user-guide). Genomes of the following related pairs were shown in Figure 3 a:) *S. halifaxensis* HAW-EB4/*S. pealeana* ATCC 700345; b) *S. sediminis* HAW-EB3 /*S. woodyi* ATCC 51908; c) *S. halifaxensis* HAW-EB4/*S. sediminis* HAW-EB3; d) *S. halifaxensis*/*S. oneidensis* MR-1. The conserved orthologous segments among bacterial pairs, referred to as Locally Collinear Blocks (LCBs) that may or may not be reordered or inverted in another genome, were identified. Same LCBs between aligned genomes were marked with the same color. Those LCBs inverted in another genome were shown on the opposite side of the axis. The minimum LCB weight was set at the lowest level to prepare the alignment results in Figure 3.

To determine the genes of *S. halifaxensis* and *S. sediminis* conserved among all 17 strains or in related strains of *Shewanella*, local reciprocal (two ways) blast (Basic Local Alignment Search Tool) 2.2.18 was used to compare the sequences of all deduced proteins in one bacterium to 16 other strains of *Shewanella* (Table 1). The computation was conducted on a station located in the Computational Chemistry and Biology group, Biotechnology Research Institute, National Research Council Canada (Montreal, Canada). The cut off E-value was set at 1e-20. The sets of CDSs that were common first hit (reciprocal blast) among all 17 strains of *Shewanella*, were identified as the core *Shewanella* CDS (CDS^c^), with the rest CDS refereed to as non-core CDSs (CDS^nc^) (Table 1). In CDS^nc^, the common first hit among *S. halifaxensis*, *S. pealeana*, *S. sediminis* and *S. woodyi* were further identified, labelled as CDS^4^, by deducting CDS^c^ from the total common first hit among *S. halifaxensis*, *S. pealeana*, *S. sediminis* and *S. woodyi*. In the rest of CDS^nc^ of *S. halifaxensis*, those common first hit with *S. pealeana* were also identified, labelled as CDS^2-hp^, by deducting CDS^c^ and CDS^4^ from the total common first hit among *S. halifaxensis* and *S. pealeana*. The remaining *S. halifaxensis* CDSs were labelled as CDS^1-ha^. The same approach was used to obtain CDS^c^, CDS^4^, CDS^2^ and CDS^1-se^ for *S. sediminis*, except that CDS^2^ was orthologs to *S. woodyi* minus those included in CDS^4^ and CDS^c^.

To clearly view the physical distribution of different sets of CDS on the chromosomes, Circular Genome Viewer software (CG view) [64] was used to generate their maps. The predicted functions of genes in each CDS set were shown by their colors, each of which represents a cog category (see legend to Fig 4a1 and b1).

To identify the close relatives of the genes in *S. halifaxensis* and *S. sediminis* and determine the source of genes of lateral transfer, different CDS sets were compared to 623 other bacterial genomes using local reciprocal (two ways) blast (Basic Local Alignment Search Tool) 2.2.18. The bacteria with the top-matched sequences were identified and considered to be the most likely sources (or origins) of the genes. For *S. halifaxensis*, the gene was considered to be foreign-originated or horizontally transferred in the following two cases. One case was, if the best-matched bacterium was not a member of *Shewanella* after excluding 1) *S. pealeana,* 2) *S. pealeana, S. sediminis* and *S. woodyi*, 3) *S. pealeana, S. sediminis*, *S. woodyi* and *S. loihica*. The second case was, if the best matched bacterium was not a member of a marine γ-proteobacteria or a γ-proteobacteria after excluding hits in *Shewanella*. The same approach was applied to identify the sources of *S. sediminis* genes except in case 1) *S. pealeana* was replaced with *S. woodyi*, and in case 2) *S. sediminis* was replaced with *S. halifaxensis*. Most of these identified horizontally transferred genes were presented on circular chromosome maps shown in Figure 4a2 for *S. halifaxensis* and b2 for *S. sediminis*. The color of the CDS on the circular map indicated the sets of CDS. The other detailed information of the CDS could be read directly online by opening the SVG format of the supplementary Figures (Fig S1, Fig S2) using Scalable Vector Graphics (SVG) viewer software. The non-*Shewanella* bacteria top-matched to CDS^c^ or CDS^nc^) were presented in Figure 5a-c.

To determine the presence or absence of a genome A gene in genome B, the protein sequence coded by the genome A gene was blasted with a cut off E-value of 1e-20 to all proteins of genome B. This gene was considered absent in genome B if no hit (or homolog) was observed (Table 3). To help visualize the presence or absence of a gene in other genomes, heatmap.2 in g-plot and R-package were also used to prepare heatmaps based on blasting results.

### Average Amino Acid Composition, GC Content, and Statistics

Perl programs written by YHD were used to calculate 1) the average composition of each amino acid (ACAA, % of total number of amino acids) in all proteins coded by total (or a subset) CDS of a bacterium (or a group of bacteria), 2) the average G+C (or A+G) content of total CDSs (or a subset) of each genome published in Genbank (dada presented in Fig 6a2 and Table 5). The deviations of ACAA for each amino acid in one bacterium (or one CDS set) were obtained by deducting the average value of the same amino acid in all bacteria (or all CDS sets) compared. The data were presented in Figure 6a1, b1, and c1. The deviation of GC content in each bacterium (or a group of bacteria, a CDS set) was calculated by deducting the average GC content of all genomes (Fig 6b2 and Fig 5c2). T-test of R package was used to calculate the difference as well as the p-value in ACAA and GC content, between the cold-adapted and warm adapted groups of marine γ-proteobacteria (or *Shewanella*) (Table 5). The cold-adapted *Shewanella* included *S. halifaxensis*, *S. pealeana*, *S. sediminis*, *S. woodyi*, and *S. frigidimarina*. The other 12 strains of *Shewanella* (as listed in Table 1) were considered as warm-adapted. A similar approach was applied to orthologous proteins (or genes) among 1) marine γ-proteobacteria, 2) 17 strains of *Shewanella*), 3) CDS^c^, CDS^4^, CDS^2^ and CDS^1^ set of *S. halifaxensis* and *S. sediminis*.

### RDX Metabolic Activities in *Shewanella* spp

*Shewanella* spp (see below) were grown for 19 h anaerobically in MB-20 medium (peptone, 16 g L^−1^; yeast extract, 4 g L^−1^; sea salts, 40 g L^−1^; pH 7.3; Tris, 20 mM) in the presence of TMAO (100 mM) as previously described [48]. Cells were harvested by centrifugation and washed twice with 4% sea salts (pH 7.2), followed by re-suspension in the same sea salts solution. The reaction solution contained 90 µM RDX, 4.8–5.8 OD_600_ of cells and a pH of 7.5 (OD_600_ of cell suspensions: *S. halifaxensis* 5.1; *S. pealeana*, 5.6; *S. sediminis*, 4.8; *S. woodyi*, 5.1; *Shewanella hanedai*, 5.8). All solutions used were degassed and placed under argon prior to use. Tests were incubated at 10°C, 150 rpm, away from light, and run in triplicate. Rates (nM h^−1^) were measured based on removal of RDX within the first 5 hours of reaction.

### *S. halifaxensis* Growth on RDX as the Sole Terminal Electron Acceptor

Growth of *S. halifaxensis* on RDX was carried out in serum bottles (60-mL, autoclaved prior to use) containing MB-20 marine medium (sterilized by passing through a 0.22 µm sterile filter membrane, 50 mL, no TMAO). RDX powder (50 mg) was added to above bottles for an oversaturated concentration of 1000 mg L^−1^. The serum bottles were sealed and made anaerobic by repeated degassing and charging with argon. *S. halifaxensis* cells pre-grown aerobically in MB-20 medium at 10°C were inoculated into above anaerobic media in the serum bottles through syringes. The initial OD_600_ in all bottles were controlled at a value close to 0.1. The cultures were incubated anaerobically at 10°C, 150 rpm, and away from light. To monitor bacterial growth, 1 mL of culture was sampled periodically in the anaerobic glove box; the samples were diluted using sterile 4% sea salts and plated on the MB-20 agar plates (MB-20 medium solidified with 15 g L^-1^ agar). The colonies were counted after 6 days of aerobic incubation at 10°C. Controls were also prepared in the same manner as described above, except no RDX was used in the medium. All tests were run in triplicate.

### RDX Reaction with Cytochrome from *S. halifaxensis* and *Saccharomyces cerevisiae*

All of the following steps for cytochrome isolation from *S. halifaxensis* were carried out in an anaerobic glove box using anaerobic solutions where applicable. The periplasmic proteins were prepared from whole cells using a protocol as described previously [59]. Total cytochromes were precipitated from 75 mL of periplasmic protein solution with 45–65% (NH_4_)_2_SO_4_ saturation [65]. The precipitate was harvested by centrifugation at 20,000 g for 30 min, and re-suspended in 7.5 mL of TDG buffer containing 20 mM Tris-HCl, 5 mM dithiothreitol (DTT) and 10% glycerol (pH 7.1). The latter solution was desalted using a PD-10 desalting column (GE Healthcare) and TDG buffer according to the manufacturer's instructions. The above suspension (1.5 mL) was separated by gravity flow on the Q Sepharose Fast Flow Resin (GE Healthcare) (7.6 mL bed volume, 15 mm in diameter×53 mm in height). Twenty four milliliters of each of the following NaCl TDG buffer solutions was used as an elutant: 0.00, 0.05 (A), 0.10 (B) and 0.20 (C), 0.30 (D), 0.40 (E) and 0.6 (F) mol L^−1^. Fractions between 4 mL and 16 mL (12 ml in total) were collected and condensed using a Microcon centrifugal filter device (Ultracel YM-10, Millipore) to 0.8 mL for subsequent analysis and RDX reaction. The SDS-PAGE analyses, heme-staining and scanning of cytochrome UV-visible spectra were conducted as previously described [59].

The above prepared cytochrome *c* and from *S. cerevisiae* (Sigma, 86% purity) were tested for RDX degradation activity under the following assay conditions. All buffer, stock solutions of RDX, cytochrome and NADH were made anaerobic by repeated degassing and charging with argon before use in the anaerobic glove box for preparation of the reaction medium. The anaerobic reaction solutions were composed of cytochrome c, NADH (Sigma, 98% purity) (1 mM), and RDX in serum bottles (6-mL) in the following amount [organism, reaction volume in µl, cytochrome concentration in mg mL^−1^, reaction time in h, initial RDX conc as mg L^−1^]: *S. cerevisiae*, 750, 0.11, 23, 26. *S. halifaxensis*, 500, 0.15, 5, 5.5. All ran in duplicate at 23°C at 90 rpm (away from light). RDX was also incubated in two controls: one contained no NADH and another contained no cytochrome. The headspace (250 µL) was sampled for N_2_O analysis by GC and the liquid phase for analysis RDX and metabolites as previously described [12].

### Proteomic Approach for Identification of *c*-Cytochrome

The SDS-PAGE band containing the 52 kDa cytochrome, once cut, was in-gel digested with Trypsin and subjected to LC-MS/MS analysis carried out in the Genome Quebec Proteomics facility. The peptide extracts were desalted on-line with a Zorbax C18 5×0.8 mm trapping column (Agilent) prior to injection onto a 10×0.75 mm Biobasic Picofrit column (New Objective). The gradient used was from 10 to 100% Solvent B (95% acetonitrile: 0.1% Formic acid) in 30 minutes. Tandem mass spectrometry analysis was done with a MicroQtof (Waters) using data directed acquisition. All MS/MS data was peaklisted with Mascot distiller v2.1 and peptide identification was carried out using Mascot v2.1 (Matrix Science, London, UK). The mascot .dat files were imported into Scaffold (Scaffold_2_02_03, Proteome Software Inc., Portland) and an X! Tandem (www.thegpm.org; version 2007.01.01.1) search was carried out. The searches were done on a subset of the NCBI nr database from 20081006 filtered for bacterial sequences (3568264 sequences). Search tolerances of 0.50 Da for the parent mass and 0.50 Da for fragment mass was set for both Mascot and X1Tandem searches. Carbamidomethylation of cysteine was set as fixed modification and oxidation of methionines as variable modification. Scaffold was used to validate MS/MS based peptide and protein identifications so as to generate a list of protein identifications that satisfy the rules of parsimony. Protein identifications were accepted if they could be established at greater than 90.0% probability as specified by the Peptide Prophet algorithm [66] and contained at least 1 identified peptide. Protein probabilities were assigned by the Protein Prophet algorithm [67].

## Supporting Information

Figure S1 *S. halifaxensis* genes of lateral transfer. The gene's function, CDS set (h1 = CDS^1^, h2 = CDS^2^, h4 = CDS^4^, h17 = CDS^c^), and most closely related non-*Shewanella* bacteria can be read by pointing mouse on any selected gene. The cog legends on the upper right corner were generated during preparation of the map and do not indicate the functions of the genes. Instead, the color of Q, P, E or I corresponds to CDS^1^, CDS^2^, CDS^4^, or CDS^c^, respectively. The light blue arrows mark genes on the reverse strand, and red arrows mark genes on forward strand of DNA.
(8.99 MB XML)

Figure S2 *S. sediminis* genes of lateral transfer. The gene's function, CDS set (h1 = CDS^1^, h2 = CDS^2^, h4 = CDS^4^, h17 = CDS^c^), and most closely related non-*Shewanella* bacteria can be read by pointing mouse on any selected gene. The cog legends on the upper right corner were generated during preparation of the map and do not indicate the functions of the genes. Instead, the color of Q, P, E or I corresponds to CDS^1^, CDS^2^, CDS^4^, or CDS^c^, respectively. The light blue arrows mark genes on the reverse strand, and red arrows mark genes on forward strand of DNA.
(9.71 MB XML)
